# The so far farthest reaches of the double jelly roll capsid protein fold

**DOI:** 10.1186/s12985-018-1097-1

**Published:** 2018-11-23

**Authors:** Carmen San Martín, Mark J. van Raaij

**Affiliations:** Departamento de Estructura de Macromoléculas, Centro Nacional de Biotecnología (CNB-CSIC), Darwin 3, 28049 Madrid, Spain

**Keywords:** Bacteriophages, Double jelly roll, Metagenomics

## Abstract

**Background:**

During the last two decades, structural biology analyses have shown that viruses infecting hosts far apart in evolution share similar architectural features, prompting a new virus classification based on structural lineages. Until recently, only a few prokaryotic viruses had been described for one of the lineages, whose main characteristic is a capsid protein with a perpendicular double jelly roll.

**Main body:**

Metagenomics analyses are showing that the variety of prokaryotic viruses encoding double jelly roll capsid proteins is much larger than previously thought. The newly discovered viruses have novel genome organisations with interesting implications for virus structure, function and evolution. There are also indications of their having a significant ecological impact.

**Conclusion:**

Viruses with double jelly roll capsid proteins that infect prokaryotic hosts form a large part of the virosphere that had so far gone unnoticed. Their discovery by metagenomics is only a first step towards many more exciting findings. Work needs to be invested in isolating these viruses and their hosts, characterizing the structure and function of the proteins their genomes encode, and eventually access the wealth of biological information they may hold.

## Structural biology and the first glimpses of the double jelly roll reach

**Fig. 1:**
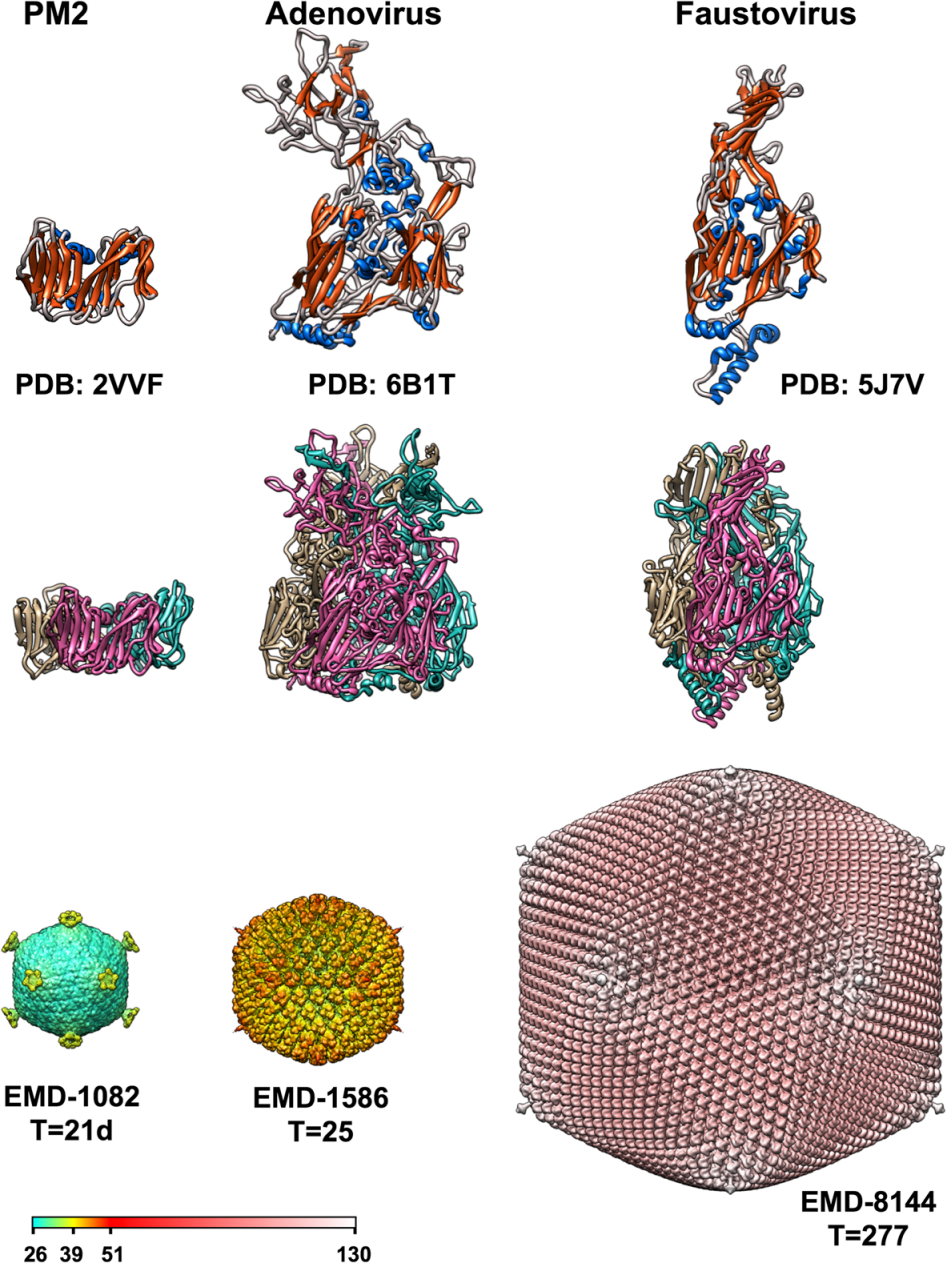
From the simplest to the most complex double jelly roll virus structures solved so far. The structures of the major capsid protein monomer (top row) and trimer (middle row) are shown, together the complete capsid (bottom row) of bacteriophage PM2, human adenovirus, and Faustovirus. These viruses represent the simplest and most complex examples for which both the high resolution structure of the major coat protein and at least the general capsid organization have been solved. While the PM2 major coat protein is formed by the double jelly roll motif with no more elaborations, the adenovirus and Faustovirus proteins have extensive tower domains which establish intricate interlacing in the trimer. Database identifiers and triangulation numbers are shown by each structure. The colour bar indicates capsid radii, in nm

Towards the end of last century, many virus structures had been determined by protein crystallography, showing that the β-barrel fold (consisting of eight antiparallel β-strands organized in two sheets that form the opposite sides of the barrel) was a common feature in the organization of icosahedral virus capsids [1]. ssDNA viruses infecting bacteria (*Microviridae* such as ΦX174), as well as ssRNA viruses infecting plants (e.g. tombusviruses), insects (tetra-, noda-, dicistroviruses), cattle (foot-and-mouth disease virus) and humans (rhinovirus, poliovirus) all were found to build their capsids using proteins that fold as a “jelly roll” β-barrel. Back then, only one dsDNA virus, human adenovirus, was known to utilize the β-barrel fold in its capsid, albeit in an odd way. The adenovirus major coat protein contains two β-barrels instead of one, an arrangement also referred to as double jelly roll [2] (Fig. 1). The adenovirus β-barrels are not parallel, but perpendicular to the capsid surface, and form pseudo-hexagonal capsomers, allowing trimeric proteins to fill in the six-fold coordinated positions of the icosahedral capsid [3].

Then, in 1999, the major coat protein structure of a peculiar, tail-less, membrane-containing dsDNA bacteriophage called PRD1 was solved, and unexpectedly proved that the human adenovirus structural solution was present also in viruses with prokaryotic hosts [4]. This finding raised questions on virus evolution, hinting at a possible common ancestor of viruses infecting prokaryotic and eukaryotic organisms [5]. At about the same time, it was also realized that herpesviruses share architectural characteristics with tailed phages, and that reoviruses have structural similarity with the bacterial cystoviruses [6, 7]. All these observations evolved into the proposal that a classification on *structural lineages*, based on major coat protein folds, might be more useful, and better reflect the evolutionary history of viruses, than previous classifications based on genome type or host [8–10].

Four icosahedral virus structural lineages are recognized at present [10], with indications that other lineages may exist, for example one encompassing positive and negative ssRNA viruses [11]. The dsDNA herpesviruses, which infect animals, form one structural lineage with tailed bacteriophages: they share many structural characteristics, including their assembly pathway and major coat protein fold. A second structural lineage includes the dsRNA cystoviruses (bacteriophages like Φ6) together with eukaryotic dsRNA viruses such as reo- or totiviruses. A third lineage encompasses picorna-like viruses, with coat proteins folding as a β-barrel lying parallel to the capsid surface. Adenoviruses, which infect vertebrates, and the tectivirus PRD1 were the founding members of the fourth icosahedral structural lineage, which encompasses dsDNA viruses infecting organisms across the evolutionary tree **(**Table 1**)**: bacteria (tectiviruses, corticoviruses), archaea (turriviruses), unicellular animals (giant viruses like mimivirus and their relatives, also their virophages) and algae (phycodnaviruses), insects, fish, amphibians and reptiles (iridoviruses), pigs (asfarviruses), and vertebrates in general including humans (adenoviruses) [10, 12]. The infectious particles of all these viruses are built from trimeric double jelly roll capsomers (Fig. 1), arranged with triangulation numbers ranging between *T* = 21 [13] and 499 [14]. The triangulation number of the giant mimivirus capsid, which has not been unequivocally determined yet, is estimated to be in the 972–1200 range [15]. Members of the double jelly roll lineage have also a single perpendicular jelly roll protein forming the pentameric vertex capsomers. Remarkably, a scaffold protein of the non-icosahedral poxviruses involved in the initial stages of assembly also folds as a double β-barrel pseudo-hexamer [16].

**Table 1:** Double jelly-roll virus families for which the major capsid protein and/or capsid structures have been studied

Example virus and family name	Host	Capsid diameter	Triangulation number	Genome type and approximate size	Ref.	Number of accepted species in ICTV [35]
Prokaryotic host - bacteria						
PRD1, *Tectiviridae*	Gram-negative bacteria	70 nm	*T* = 25	linear dsDNA, 15 kbp	[36]	6
PM2, *Corticoviridae*	*Pseudoalteromonas*	60 nm	T = 21*d*	circular dsDNA, 10 kbp	[33]	1
*Salisaeta* Icosahedral phage 1 (SSIP-1), *Sphaerolipoviridae?,* Unclassified	*Salisaeta sp*	100 nm (single jelly roll?)	*T* = 49	circular dsDNA, 44 kbp	[37]	–
Flavobacterium-infecting, lipid-containing phage (FLiP), Unclassified	*Flavobacterium sp.*	55 nm	T = 21*d*	circular ssDNA, 9 kb	[19]	–
Prokaryotic host - archaea						
STIV, *Turriviridae*	*Sulfolobus solfataricus*	96 nm (with turrets) 73 nm (without)	*T* = 31*d*	circular dsDNA, 18 kbp	[38]	2
HHIV-2, *Sphaerolipoviridae*	*Haloarcula* *hispanica*	80 nm (single jelly roll)	*T* = 28*d*	linear dsDNA, 30 kbp	[22]	7^a^
Eukaryotic host						
Paramecium bursaria chlorella virus 1 (PBCV-1), *Phycodnaviridae*	*Chlorella variabilis*	190 nm	*T* = 169*d*	dsDNA with covalently closed hairpin termini, 330 kbp	[39, 40]	33
*Phaeocystis pouchetii* virus 1 (PpV01) *Phycodnaviridae* or *Mimiviridae*: under debate.^b^	*Phaeocystis pouchetii* (phytoplankton)	220 nm	*T* = 219	485 kbp	[40, 41]	–
Cafeteria roenbergensis virus (genus Cafeteriavirus, *Mimiviridae*)	*Cafeteria roenbergensis* (zooplankton)	300 nm	*T* = 499	730 kbp	[14, 42]	1^c^
*Acanthamoeba polyphaga* Mimivirus (APMV), genus Mimivirus, *Mimiviridae*	*Acanthamoeba polyphaga*	500 nm	*T* = 972–1200	linear dsDNA, 1180 kbp	[15, 43]	1
Sputnik, *Lavidaviridae*	Amoebae/Mimivirus (virophage)	75 nm	*T* = 27	circular dsDNA, 18 kbp	[44]	3
Melbournevirus, *Marseilleviridae*	*Acanthamoeba castellanii*	230 nm	*T* = 309	circular (?) dsDNA, 369 kbp	[45, 46]	4
Faustovirus, unclassified (distantly related to *Asfarviridae*)	*Vermamoeba vermiformis*	260 nm	*T* = 277	circular dsDNA, 466 kbp	[47–49]	–
Pacmanvirus, unclassified (distantly related to Faustovirus and *Asfarviridae*)	*Acanthamoeba castellanii*	250 nm	T = 309	dsDNA, 395 kbp	[50]	–
Chilo iridescent virus (CIV), *Iridoviridae*	Invertebrates, amphibians, fish	185 nm	*T* = 147	linear dsDNA, 212 kbp	[51, 52]	13
Adenovirus, *Adenoviridae*	Vertebrates	95 nm	T = 25	linear dsDNA, 27–43 kbp	[3]	104
Vaccinia virus, *Poxviridae*	Vertebrates	200–300 nm	Non-icosahedral	linear dsDNA, 130–375 kbp	[16]	71

^a^Two of these seven sphaerolipovirus species have been isolated from extremophile bacterial hosts [21]

^b^For a recent discussion on the diversity of Mimiviridae and their taxonomic challenge, see Ref. [53]

^c^Claverie and Abergel [53] list eleven members of the Mimiviridae family that have been physically isolated and fully sequenced, covering a genome length range of 370–1500 kbp and a particle size range of 140–600 nm (for the icosahedral shell)

## How did the double jelly roll fold jump from prokaryotic to eukaryotic hosts?

The fact that viruses with different hosts share a common structural solution suggests that the architecture was established in the early stages of evolution, before the branches of the evolutionary tree diverged into the three kingdoms known today (archaea, bacteria and eukarya). Intriguingly, an evolutionary connection has been found between viruses in the double jelly roll lineage and large (15–20 kbp) eukaryotic double-stranded DNA transposons called Polintons [17]. Polintons are so named because they all encode a protein-primed DNA polymerase (to sustain self-replication, POL) and a retroviral-like integrase (INT). Most of them also include genes for a DNA-packaging ATPase and a maturation protease like those found in double jelly roll lineage viruses. Exhaustive sequence analyses revealed that these transposable elements also encode genes that could translate into double or single jelly roll proteins, suggesting that at some point in time, or in certain conditions, they could form icosahedral capsids.

In the light of all these findings, an evolutionary model was proposed in which a primordial, PRD1-like double jelly roll phage (encoding a double jelly roll capsid protein, a protein-primed DNA polymerase and a packaging ATPase) would have invaded a proto-eukaryotic host with a bacterial endosymbiont (mitochondria), somehow reached the nucleus, and recombined with a eukaryotic transposable DNA element carrying the integrase and maturation protease. This “polintovirus” element would have then evolved in separate ways to produce the polintons (transposable, capsid-less integrating elements), and a variety of eukaryotic “free-standing” viruses, all the way from adenovirus to mimiviruses [18].

## New findings from metagenomics extend the double jelly roll reach

The great majority of known dsDNA viruses belong to either the tailed phage/herpes lineage or to the double jelly roll lineage. The tailed phage/herpes lineage is massively dominated by the tailed phages, with herpesviruses the only eukaryotic members. Conversely, there is a large variety of double jelly roll viruses infecting eukaryotic hosts, from algae to humans, while only a few lineage members with prokaryotic hosts (bacteria and archaea) have been isolated **(**Table 1**)**. Even within this paucity, some discoveries hinted at variant uses of the double jelly roll architecture, and its possible widespread use in the prokaryotic world. On the one hand, the *Flavobacterium*-infecting, lipid containing phage FLiP, has a double jelly roll architecture but a circular ssDNA instead of a dsDNA genome [19], demonstrating the use of similar architectural solutions irrespective of genome nature. On the other, some viruses infecting archaea or extremophile bacteria encode two major coat proteins, each folding as a single β-barrel, that combine in hetero-multimers to produce capsids with the single jelly rolls perpendicular to the surface [20–22]. The existence of these later viruses supports the hypothesis that double jelly roll coat proteins may have evolved from single jelly rolls by gene duplication [23].

Progress in structural biology technologies facilitated the studies on large, complex coat proteins and virus particles that were instrumental in revealing the structural lineages. In parallel, highly advanced DNA sequencing methods became common, paving the way for environmental metagenomics projects that are nowadays the main source of virus discovery [24, 25]. Metagenomics allows virus discovery even if the host is not known or cannot be cultured in laboratory conditions. By providing previously inaccessible, large amounts of sequence data, metagenomics has also facilitated the analysis of virus evolution trends. Marine metagenome analyses have recently revealed a new group of putative polinton-like viruses in algae [26]. Polinton-like virus genomes contain genes for single and double jelly roll proteins and a packaging ATPase, but lack the protease and integrase genes. Therefore, polinton-like viruses could represent a minimal version of the double jelly roll lineage in eukaryotic hosts, or perhaps the first eukaryotic dsDNA viruses to evolve from bacterial ancestors [26].

Morphological surveys on marine samples suggested that non-tailed phages might even be more abundant than the tailed ones, despite their scarcity in culture and sequence collections [27]. More recently, examination of agents infecting marine *Vibrionaceae* bacteria has revealed that a new group of double jelly roll viruses, the autolykiviruses, has a very broad host range, and may be responsible for a large part of deaths in marine bacteria, indicating the ecological relevance of double jelly roll tail-less phages [28, 29]. With 10 kbp long genomes and 49 nm diameter capsids, the autolykiviruses would be the smallest members of the double jelly roll lineage found so far.

A more recent study used the previously identified prokaryotic double jelly roll major coat protein sequences as bait for mining the GenBank and metagenomics databases [30]. Some of the hits found were flanked by typical bacterial genes, reminding us that analyses limited to genomic sequences might identify non-functional prophages as well as actual viruses. But once this was taken into account, the authors found indications that many more double jelly roll virus families may exist in the prokaryotic landscape, including a completely new group of viruses (termed Odin), which has no characterized members. It was remarkable that, when the database search was carried out with just the presence of the double jelly roll major coat protein as a common trait, a large variety of genome organizations was found. It was observed that two genes previously thought to be fundamental lineage traits can be absent: the protein-primed replication polymerase, and the packaging ATPase. These were considered part of the “primordial” double jelly roll virus in bacteria that recombined with transposons in eukaryotic cells [18]. The finding that double jelly roll prokaryotic viruses may exist without these two genes raises questions about their mode of assembly and replication, and their place in the evolutionary landscape.

The role of the packaging ATPase is still a mystery for many double jelly roll viruses. While it seems to function as a bona fide portal for genome translocation into a preformed capsid in bacteriophage PRD1 [31, 32], such a function does not appear so obvious for members of the lineage where topological constraints are at odds with genome translocation. For example, it is not clear how the corticovirus PM2, with its circular, supercoiled dsDNA genome, or adenovirus, with a linear dsDNA genome heavily covered by protein, would use a portal with a packaging ATPase for genome translocation [13, 33, 34]. Until recently, only FLiP, the single lineage member with a circular ssDNA genome, had been found to lack the ATPase gene [19]. Now it is found that viruses in the Odin group also lack it, and have instead an open reading frame coding for a small protein preceding the major coat protein gene. This small protein has no detected similarity to any known proteins, but is conserved throughout the group.

## Conclusions

Prokaryotic double jelly roll viruses are much more abundant and hold much more genomic variability than previously thought. These realizations open the way to exciting future findings: more new viruses, new modes of genome replication and particle assembly, new host-pathogen interactions, and ecological relevance. To achieve all this new knowledge, several steps need to be addressed first, such as identifying the virus hosts, isolating the virus particles themselves, solving the structure of the capsid and determining the folds of other virus protein structures.
